# Endogenous Heparinoids May Cause Bleeding in Mucor Infection and can be Detected by Nonactivated Thromboelastometry and Treated by Recombinant Activated Factor VII: A Case Report

**DOI:** 10.1097/MD.0000000000002933

**Published:** 2016-03-03

**Authors:** Miroslav Durila, Petr Pavlicek, Ivana Hadacova, Jiri Nahlovsky, Daniela Janeckova

**Affiliations:** From the Department of Anesthesiology and Intensive Care Medicine, Second Faculty of Medicine, Charles University and University Hospital Motol (MD, PP), Department of Clinical Haematology, Motol University Hospital (IH), Department of Paedriatric Surgery (JN), and Department of Paediatric Haematology and Oncology, Second Faculty of Medicine, Charles University and University Hospital Motol, Prague, Czech Republic (DJ).

## Abstract

Mucormycosis is an aggressive fungal infection, which invades endothelial cells of blood vessels. This condition might lead to destruction of endothelium and release of heparin-like substances to the bloodstream and cause life-threatening bleeding, which is not well described in the literature.

We present a patient with mucormycosis who experienced life-threatening bleeding, although no standard laboratory test could detect any coagulopathy.

The cause of bleeding-coagulopathy was detected only by nonactivated thromboelastometry (NATEM), which revealed the presence of heparin-like substances. After treatment with recombinant activated FVII rotational thromboelastometry, results improved and the patient stopped bleeding. Regular application of the drug was necessary during acute phase of infection to prevent further bleeding.

In this case report, we show that NATEM can detect the presence of heparin-like substances in bleeding patient with mucormycosis infection and that recombinant activated FVII can be used to stop and prevent bleeding until infection resolves.

## INTRODUCTION

Thromboelastometry is increasingly used in coagulation monitoring of bleeding patients in intensive care unit. It is a global coagulation test, which analyses coagulation properties of the whole blood and gives us information about all phases of coagulation process including initiation phase (CT [clotting time], pathology indicates deficiency of coagulation factors or heparin effect), propagation phase (clot formation time and alpha angle, is pathologic in case of thrombocytopenia/pathia or low fibrinogen level), maximum strength of coagulum (MCF [maximum clot firmness], is pathologic in case of thrombocytopenia/pathia or low fibrinogen level), and fibrinolysis (LI 30 [lysis index], which describes percentage of remaining clot stability in relation to the MCF value at 30 min after CT). Another test such as FIBTEM (reagent contains cytochalazin D-inhibitor of platelets) gives us information about the level of functional fibrinogen and HEPTEM (contains heparinase) gives us information of the heparin presence in the blood. EXTEM is a test that provides information about extrinsic pathway of coagulation and INTEM about intrinsic pathway. NATEM test is the most sensitive to the coagulopathy, but is not routinely used because the blood sample needs some time for stabilization before analysis (citrate and calcium interaction is timely dependent).^1,2^

However, in practice, the patient is sometimes bleeding despite normal values of activated types of exams such as EXTEM and INTEM, as we also described in our recently published case report wherein NATEM showed coagulopathy despite normal EXTEM/INTEM in a bleeding patient.^3^

We present a case wherein the NATEM could help in diagnosis of heparin-like substances despite normal values of EXTEM/INTEM in a patient with mucormycosis and this was effectively treated with recombinant activated factor VII. This is not well described in the literature.

### Presenting Concerns

The subject of this report is a 12-year-old girl (58 kg, 160 cm, no bleeding disorder in anamnesis) who was admitted to the Department of Infectious Diseases with the history of 4 days lasting gastrointestinal symptoms (vomiting, diarrhea), fever, headache, and pain in upper right epigastrium. Laboratory investigation revealed positive serology for hepatitis A virus (IgM) and for Coxsackie virus. Because of ascites and fluidothorax, she underwent drainage of thorax and abdomen with iatrogenic spleen puncture and subsequent severe bleeding into the abdomen. This was complicated by mucormycosis infection of the abdomen.

### Clinical Findings

Because of abdominal bleeding, splenectomy was performed but the bleeding continued. Another surgical revision of the abdomen for suspected abdominal bleeding was done twice, but no source of bleeding was found. During the next days, she continued to bleed into abdominal drains and everyday she needed transfusion of red blood cells, fresh frozen plasma, and platelets. Surgical source of bleeding was excluded by computed tomography angiography. Timeline of diagnostic and therapeutic procedures are summarized in Table 1.

**TABLE 1:**
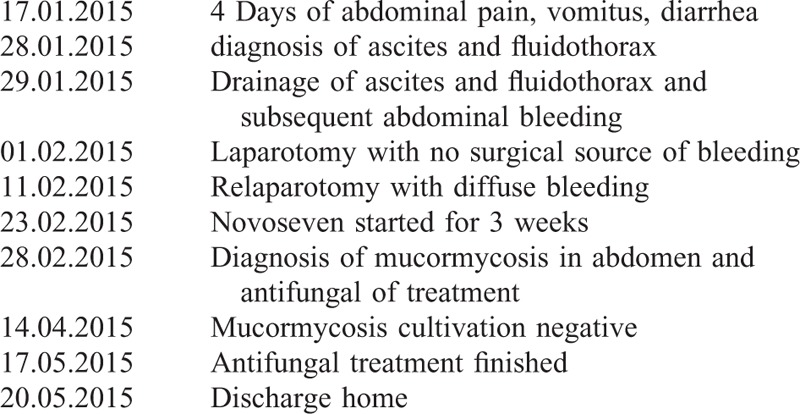
Timeline

### Diagnostic Focus and Assessment

**FIGURE 1:**
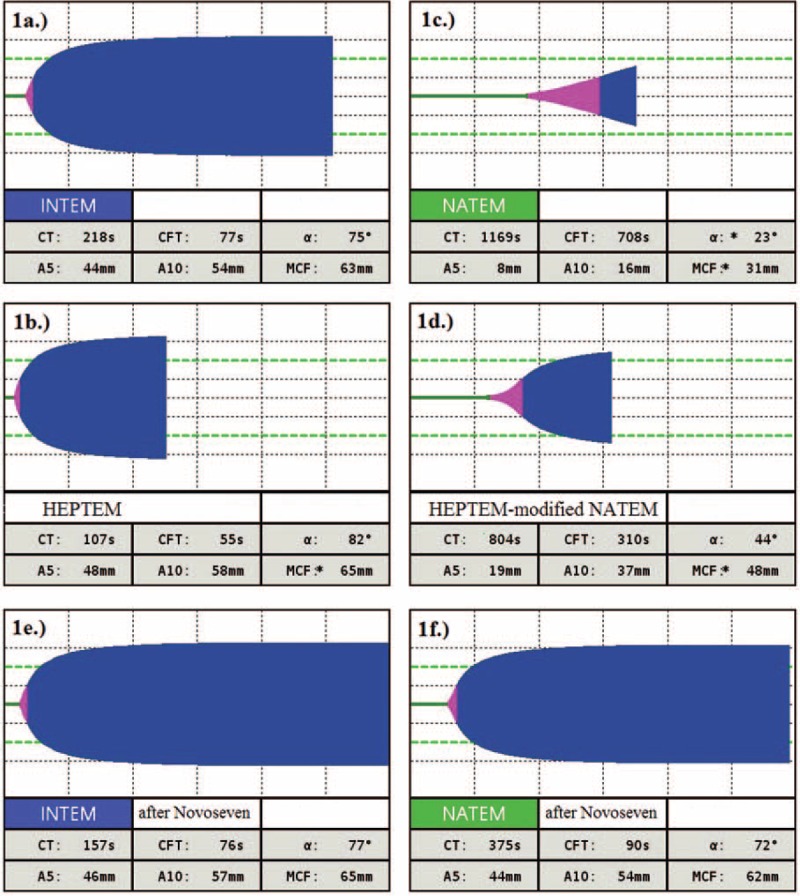
Thromboelastometry curves in the presence of heparin-like substances and after treatment of rFVIIa. (A) INTEM; (B) HEPTEM; (C) NATEM; (D) HEPTEM-modified NATEM (after adding HEPTEM reagent-containing heparinase, into the cuvette during performing control, HEPTEM-modified NATEM, test); (E) INTEM after Novoseven treatment; (F) NATEM after Novoseven treatment.

Except for the activated partial thromboplastin time ratio, which was slightly prolonged to ratio 1.39, standard coagulation tests were almost normal: prothrombin time ratio 1.0, normal level of fibrinogen (3.34 g/L), platelets count of 75 × 10^9^ cells/L and also no pathology in individual clotting factors. When activated ROTEM (EXTEM, INTEM) was performed, there was no pathology in any phase of coagulation, including overt fibrinolysis. Because the patient was still bleeding, despite of normal EXTEM and INTEM results, we decided to use a nonactivated method (NATEM), which showed pathologic prolongation of coagulation time (with no clot in the cuvette), which improved after adding heparinase (using HEPTEM reagent—containing heparinase and calcium, which was added to the cuvette instead of STARTEM reagent during performing control, HEPTEM-modified NATEM, test), and thus presence of heparin-like substances was suspected (Figure 1C and D). Afterwards, HEPTEM was performed to compare it with INTEM as well, confirming the presence of heparinoids (Figure 1A and B).

### Therapeutic Focus and Assessment

Prothamin sulphate was administered to the patient but with no effect on bleeding. As recommended by the haematologist recombinant activated FVII (rFVIIa, Novoseven, Novo Nordisk, Denmark®) treatment was started, 1 mg every 6 hours. Immediately, the patient stopped bleeding and NATEM and INTEM results improved (Figure 1E and F). After a few days, the presence of *Rhizopus microsporus* was identified in abdominal drains and in a smear from the abdominal wound and antifungal therapy was initiated.

### Follow-up and Outcomes

The treatment with rFVIIa was necessary for as long as 21 days (the bleeding reoccurred every time the treatment was stopped). Although the fungal infection was positive in smears for 48 days, during the period of treatment with Novoseven and also after 3 weeks of antifungal treatment, surgical interventions (that were needed daily for bandage changes) could be done safely without bleeding complications. Primary cause of the patient's disease was not clear, but (probably secondary) fungal infection leads to critical bleeding, which threatened the patient's life.

## DISCUSSION

Mucormycosis is a severe fungal infection, which is caused by *Rhizopus microsporus*. Extensive angioinvasion destroying vascular endothelial cells is characteristic for the course of the disease.^4^ Endothelial matrix contains endogenous heparinoids (heparin sulphate, heparin and dermatan sulphate), which can be released into bloodstream as detected by thromboelastography^5,6^ and owing to their anticoagulant effect cause severe bleeding, which can resolve only after successful treatment of the infection.^7^

To get fast results of thromboelastometry, activators of coagulation are used in INTEM (intrinsic pathway activator) and EXTEM (extrinsic pathway of activation) tests. However, as we have showed at our previous case done on thromboelastography, artificial activators can modify final results.^8^ In practice, the patient is sometimes bleeding despite the normal result of EXTEM and INTEM. In our case, we show that NATEM can be helpful in detection of heparin-like substances present in mucormycosis infection ,which were also confirmed by comparing normal INTEM to HEPTEM. After rFVIIa treatment, although Novoseven treatment in this case was an off-label use of the drug, the patient stopped bleeding and thromboelastometry results improved. The treatment was needed until the acute stage of infection resolved and thus we can speculate that during this period the endothelium restored and endogenous heparin-like substances disappeared.

In this case report, we want to show that NATEM should be used in a bleeding patient when EXTEM and INTEM are in normal ranges and that heparin-like substances may cause severe bleeding in mucormycosis. The bleeding patient can be saved with rFVIIa treatment until the infection resolves and vascular endothelium restores.

### Informed Consent

Patient and her parents gave consent with publication of the case.
